# Moving towards precision psychiatry: the hard nut of depression

**DOI:** 10.1038/s41392-024-02023-8

**Published:** 2024-11-07

**Authors:** Juergen Dukart, Leon D. Lotter, Simon B. Eickhoff

**Affiliations:** 1 Institute of Neurosciences and Medicine, Brain & Behaviour (INM-7), Research Centre Juelich, Juelich, Germany; 2 Institute of Systems Neuroscience, Medical Faculty, Heinrich Heine University, Düsseldorf, Germany; 3 Max Planck School of Cognition, Leipzig, Germany

**Keywords:** Predictive markers, Diseases of the nervous system

In a recent study published in *Nature*, Lynch et al.^1^ reported a consistent spatial expansion of the resting state salience network in subjects with major depression. The expansion was already present in children who later developed depression and remained stable over time in patients with depression, indicating a trait-like behavior of the observed endophenotype.

Research on major depression is symbolic of the challenges faced in the study of most, if not all, psychiatric disorders. Whilst various therapeutic options exist, about a third of patients with major depression do not respond to existing treatments, and no validated biomarkers exist for early diagnosis, prognosis, or monitoring of the disease. Naturally, for a brain disease, neuroimaging is often the method of choice in studies aiming to identify biomarkers for depression with, to date, very modest results. Brain alterations in depression were either reported to be of small effect size and therefore not clinically meaningful, or they consistently failed to replicate in later independent cohorts. Similarly, neuroimaging-based machine learning models have continuously yielded close to chance-level accuracies for differentiating between major depression patients and healthy controls in large independent cohorts.^2^

Instead of looking at depression-related brain alterations using classical region- or voxel-wise statistics, Lynch et al. adopted a network-centered perspective comparing the topology and spatial extent of well-established resting state connectivity patterns between patients and healthy controls. Using this approach, the authors report the intriguing finding of a spatially expanded salience network with very large effect sizes observed in the deeply-phenotyped high-density longitudinal discovery cohort. The authors then replicate this finding in two independent cohorts with still large, although substantially reduced, effect sizes. The salience network is well established in the literature and likely operates under the substantial influence of noradrenergic and mesolimbic dopaminergic inputs. Functionally, it is implicated in conscious integration of autonomic feedback with internal goals as well as social-emotional regulation.^3^ These functions align well with the symptomatology observed in depression, adding face-validity to the findings by Lynch et al.

The adopted topological approach also illustrates the necessity of moving away from classical voxel- or region-wise inferential statistics towards more complex methods such as taking into account the spatial topology of respective neuroimaging measures. Despite the promising findings by Lynch et al., the biological factors that shape network topology, as well as the pathophysiological mechanisms underlying the observed expansion of the salience network in major depression, remain elusive. Understanding both is essential for development of accurate diagnostic tools and improved interventions. To address the limited insight into the underlying pathophysiological mechanisms, the applied topology methods can now be combined with publicly available gene expression and neurotransmitter atlases as well as with other neurophysiological modalities.^4^ Integrating this multimodal information would greatly facilitate the interpretation of the pathophysiological mechanisms underlying the respective findings and allow for more guided hypothesis generation regarding potential interventions (Fig. 1).

**Fig. 1:**
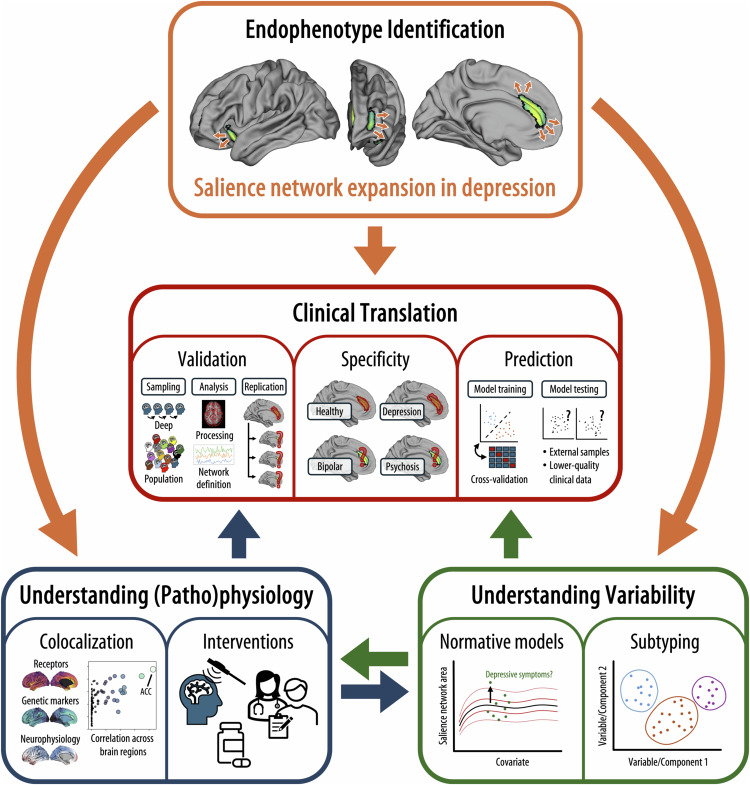
Conceptual overview of the next steps for translation of the findings by Lynch et al.^1^ into clinical applications for major depression. To enable clinical translation, the observed endophenotypes need to be integrated with other neurophysiological modalities to gain insights into the pathomechanisms underlying the network expansion observed in major depression. Normative modeling and subtyping studies are further required to gain insights into the variability and reliability of expansion endophenotypes in the general as well as in the major depression population. Both needs to be combined with prospective clinical studies to validate the findings as potential biomarkers for diagnosis and prognosis of major depression

Considering the trait-like behavior of the identified network expansion, normative modeling may be a further promising avenue to gain a better understanding of the variability of this endophenotype in the general population. In this regard, follow-up research will also have to explore the specificity of the observed salience network expansion to major depression. Both are important prerequisites for a biomarker to be considered for clinical applications. For the first, healthy control populations recruited into clinical studies are often highly selective. Such preselection may have narrowed the actual variability in network expansion in the control cohort and thereby inflated the observed effect sizes. For the second, many of the endophenotypes reported in the literature for one psychiatric disorder are often later rediscovered in other psychiatric disorders. Understanding the specificity of the observed expansion effects is therefore important for assessing the actual diagnostic or predictive value of these endophenotypes. Similarly, a crucial part of future research with respect to the observed network expansion needs to be directed towards understanding the high variability in clinical symptomatology and treatment response observed in major depression. Understanding if and how the observed expansion aligns with potential depression subtypes and relates to treatment effects at the individual level will be essential for moving the findings into clinical applications.

On a more cautious note, the authors trained a machine learning-based classification framework using the extracted network expansion features. They report rather high accuracies of about 78% for differentiation between depression cases and healthy controls, with salience network expansion unsurprisingly providing the largest contribution. Despite these promising findings, it is important to note that the search for machine learning biomarkers in psychiatry has been continuously hampered by failures to replicate and generalize. For example, several promising machine learning models for schizophrenia diagnosis initially performed well in large multi-centric datasets, but all failed to replicate in truly independent cohorts.^5^ Small sample sizes, study-specific confounding effects such as differences in demographics or acquisition parameters, and processing and analyses choices contributed to limited generalization of previous machine learning models. Several of these limitations including the limited sample size and specific processing and analysis choices certainly also apply to the study by Lynch et al. In this respect, whilst optimism is warranted, a prospective and independent replication of the findings is imperative for further pursuit into clinical translation.

The innovative approach introduced by Lynch et al. certainly provides a promising path to study psychiatric disorders and, more specifically, to translate neuroimaging measures into clinically relevant biomarkers for major depression. In this regard, the demonstrated applicability to individual patients indicates its high potential for moving the field towards true precision psychiatry.

## References

[1] Lynch, C. J. et al. Frontostriatal salience network expansion in individuals in depression. *Nature* **633**, 624–633 (2024). 39232159 10.1038/s41586-024-07805-2 PMC11410656

[2] Winter, N. R. et al. A systematic evaluation of machine learning–based biomarkers for major depressive disorder. *JAMA Psychiatry* **81**, 386–395 (2024). 38198165 10.1001/jamapsychiatry.2023.5083 PMC10782379

[3] Seeley, W. W. The salience network: a neural system for perceiving and responding to homeostatic demands. *J. Neurosci.* **39**, 9878–9882 (2019). 31676604 10.1523/JNEUROSCI.1138-17.2019 PMC6978945

[4] Lotter, L. D. et al. Regional patterns of human cortex development correlate with underlying neurobiology. *Nat. Commun.* **15**, 7987 (2024). 39284858 10.1038/s41467-024-52366-7 PMC11405413

[5] Chekroud, A. M. et al. Illusory generalizability of clinical prediction models. *Science* **383**, 164–167 (2024). 38207039 10.1126/science.adg8538

